# Altered Relaxation and Mitochondria‐Endoplasmic Reticulum Contacts Precede Major (Mal)Adaptations in Aging Skeletal Muscle and Are Prevented by Exercise

**DOI:** 10.1111/acel.70137

**Published:** 2025-06-30

**Authors:** Ryan J. Allen, Ana Kronemberger, Qian Shi, R. Marshall Pope, Elizabeth Cuadra‐Muñoz, Wangkuk Son, Long‐Sheng Song, Ethan J. Anderson, Renata O. Pereira, Vitor A. Lira

**Affiliations:** ^1^ Department of Health and Human Physiology, Fraternal Order of Eagles Diabetes Research Center, College of Liberal Arts and Sciences University of Iowa Iowa City Iowa USA; ^2^ Department of Internal Medicine, Fraternal Order of Eagles Diabetes Research Center, Carver College of Medicine University of Iowa Iowa City Iowa USA; ^3^ Proteomics Facility, Carver College of Medicine University of Iowa Iowa City Iowa USA; ^4^ Department of Pharmaceutical Sciences & Experimental Therapeutics, College of Pharmacy, Fraternal Order of Eagles Diabetes Research Center University of Iowa Iowa City Iowa USA

**Keywords:** aging, endoplasmic reticulum, exercise, mitochondria, mitochondrial‐associated ER membranes, sarcopenia, sarcoplasmic reticulum, skeletal muscle

## Abstract

Sarcopenia, or age‐related muscle dysfunction, contributes to morbidity and mortality. Besides decreases in muscle force, sarcopenia is associated with atrophy and fast‐to‐slow fiber type switching, which is typically secondary to denervation in humans and rodents. However, very little is known about cellular changes preceding these important (mal)adaptations. To this matter, mitochondria and the sarcoplasmic reticulum are critical for tension generation in myofibers. They physically interact at the boundaries of sarcomeres, forming subcellular hubs called mitochondria‐endo/sarcoplasmic reticulum contacts (MERCs). Yet, whether changes at MERCs ultrastructure and proteome occur early in aging is unknown. Here, studying young adult and older mice, we reveal that aging slows muscle relaxation, leading to longer excitation‐contraction‐relaxation (ECR) cycles before maximal force decreases and fast‐to‐slow fiber switching takes place. We also demonstrate that muscle MERC ultrastructure and mitochondria‐associated ER membrane (MAM) protein composition are affected early in aging and are closely associated with the rate of muscle relaxation. Additionally, we demonstrate that regular exercise preserves muscle relaxation rate and MERC ultrastructure in early aging. Finally, we profile a set of muscle MAM proteins involved in energy metabolism, protein quality control, Ca^2+^ homeostasis, cytoskeleton integrity, and redox balance that are inversely regulated early in aging and by exercise. These may represent new targets to preserve muscle function in aging individuals.

## Graphical abstract

Slowed relaxation and altered MERCs embody early stages of aging in muscle, and are prevented by regular exercise.

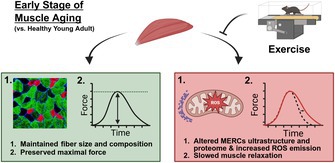

## 1 Introduction

By 2050, individuals aged 60 years or older will represent over 20% of the global population (Chatterji et al. 2015). As this demographic expands, so do the inevitable ailments accompanying biological age. One such condition is sarcopenia, defined as the progressive and generalized loss of muscle mass and function with age, which reportedly affects up to 29% of older individuals (Cruz‐ Jentoft et al. 2014; Lang et al. 2010). Sarcopenia not only detracts significantly from quality of life but also markedly increases risks of morbidity and mortality (Budui et al. 2015). Therefore, healthcare efforts to attenuate the progression of sarcopenia (herein referred to as age‐related muscle dysfunction) are critical for improving the health span of our aging population. However, effective therapies remain limited because our understanding of the structural and functional events occurring early in muscle aging remains scarce.

It is well documented that muscle maximal force decreases dramatically in advanced ages (Doherty 2003; Lang et al. 2010; Sayer et al. 2024). At the cellular level, these functional changes are accompanied by pronounced atrophy of type 2 fibers and involve fast‐to‐slow (i.e., type 2 to type 1) fiber type switching, which is typically secondary to type 2 fiber denervation (Aagaard et al. 2010; Dowling et al. 2023; Lang et al. 2010; Larsson et al. 2019). However, very little is known about functional changes preceding these important (mal)adaptations. Though it is well recognized that regular exercise alleviates the impact of aging on skeletal muscle, exercise‐mediated changes that occur early in aging are not well‐characterized (Budui et al. 2015; Cartee et al. 2016; Schumann et al. 2022; Wang et al. 2022).

Both the sarcoplasmic reticulum (SR), a specialized form of the endoplasmic reticulum (ER) in muscle, and mitochondria are required for tension generation in skeletal myofibers. Mitochondria‐endo/sarcoplasmic reticulum contacts (MERCs), also known as mitochondria‐associated ER/SR membranes (MAMs), are sites of physical coupling between the outer mitochondrial membrane (OMM) and the ER/SR. Across cell populations, MERCs modulate numerous processes such as ion and nucleotide exchange, lipid metabolism, and autophagosome formation, among others (Giacomello and Pellegrini 2016; Hamasaki et al. 2013). Within muscle, MERCs are predominately located at sarcomeres near the intersection of the I‐band and the Z‐disc. Because mitochondria and the ER/SR become dysfunctional in sarcopenic muscle and adapt to exercise, MERCs likely play a role in mediating these changes (Baehr et al. 2016; Bohnert et al. 2018; Kauppila et al. 2017; Kubat et al. 2023; Paez et al. 2023; Parry et al. 2020; Tarnopolsky et al. 2007) In fact, recent studies have documented irregular MERC/MAM morphology and protein composition in skeletal muscle across multiple conditions (Hinton Jr. et al. 2024; Lu et al. 2022; Thoudam et al. 2018; Tubbs et al. 2018).

In this study, we reveal that early muscle aging is characterized by slowed relaxation and increased fatigue. This occurs before reductions in muscle force or changes to fiber type and is effectively reversed by regular exercise. Additionally, we demonstrate that muscle MERC/MAM ultrastructure and protein composition are affected at this stage and are closely associated with the changes to muscle relaxation. Moreover, we reveal for the first time a set of muscle MAM proteins that are inversely regulated by aging and exercise, offering novel insights into potential therapeutic targets to preserve or rescue muscle function in our aging population.

## 2 Experimental Procedures

### 2.1 Animals

All experimental procedures were conducted using male, C57BL6N mice and were approved by the University of Iowa Institutional Animal Care and Use Committee. Animals were housed onsite (Medical Laboratories Vivarium, University of Iowa, Iowa City, IA) in 21°C controlled rooms operating on 12‐h light–dark cycles. Mice had ad‐lib access to water and Teklad global soy protein‐free, irradiated diet at all times (2920×). Our experimental groups consisted of young (HYA, 6 months), old (eAMD, 21 months), and very old (aAMD, 31 months) animals. Of note, the very old animals considered in this study were tumor‐free. The exercise group (eAMD+Ex) started training at 19 months of age, finishing the intervention at the same age as eAMD. Euthanasia was performed via cervical dislocation following anesthetization by 3% isofluorane gas. Hindlimb muscles were harvested at the beginning of the light cycle at least 36 h after the last bout of exercise.

### 2.2 In Vivo Muscle Function

Set‐up, assessment, and analysis of muscle force using the 1300A 3‐in‐1 Whole Animal System (1300A; Aurora Scientific, Aurora, ON, Canada) was performed as previously described (Fuqua et al. 2019). All measurements were performed via percutaneous stimulation of the tibial nerve to elicit isometric plantar flexor contraction. Anesthetized under 3%–4% isoflurane, each animal was first subjected to a maximal stimulus (150 Hz), and then the fatigue protocol was initiated after a 90‐s rest period. The fatigability protocol includes 70 submaximal tetanic contractions (50 Hz) with 5 s between each. This stimulation frequency (50 Hz) was chosen because it elicits ~ 60%–70% of maximal force, which is closer to daily contractions. Percentage of force preservation was calculated by using the mean of the last 5 contractions as a percentage of the first 5 contractions during the fatigue test. The DMA software automatically reports other variables (i.e., max rate of contraction/relaxation), and these were obtained and reported from the maximal (i.e., 150 Hz) stimulations. Plantarflexor (gastrocnemius (GA), soleus (SOL), and plantaris (PL)) and dorsiflexor (tibialis anterior (TA) and extensor digitorum longus (EDL)) muscles were harvested no earlier than 48 h following the last functional test.

### 2.3 Treadmill Intervention Protocol and Exercise Capacity Testing

The 6–8 week intervention was performed on a treadmill (Panlab/Harvard Apparatus 5‐lane Touchscreen Treadmill) using a separate group of age‐matched eAMD mice (i.e., regular exercised from 19 to 21 months of age). Training began at a 5% incline and speed of 12 m/min (i.e., ~ 50% VO_2_max (Schefer and Talan 1996)) for 25 min. Animals were trained 5 days per week at the beginning of the light cycle as recently described (Schenk et al. 2024). Running time was increased by one minute each session to a maximum of 50 min, and speed was increased by 2 m/min every week. All sessions after the first week were at a 10% incline. Fitness levels were maintained by using the volume of the final training day until the completion of post‐intervention testing. Tissues were harvested no earlier than 48 h following the last bout of exercise.

Treadmill exhaustion tests were performed to quantify exercise capacity. Baseline and control animals were given a 3‐day acclimation period of 6 m/min for 10 min. Following a 5‐min warm‐up at 6 m/min, the exhaustion protocol began at 7.2 m/min with a 5% incline and acceleration of 0.27 m/min until failure. Mice were considered exhausted after falling three times onto the electric grid at the rear portion of the treadmill within 10 s. Total work capacity was calculated and reported based on running distance and body weight.

### 2.4 Mitochondrial O_2_ Consumption, ATP Production, and H_2_O_2_ Emission in Permeabilized Fibers

Preparation and handling of saponin‐permeabilized mouse red GA fibers has been previously described in detail (Lark et al. 2016). Additional information on buffers and reagents can be found within the Data S8. Both ATP production and high‐resolution respirometry were conducted using the Oroboros Oxygraph‐2 k with a previous protocol from our group (O_2_k; Innsbruck, Austria) (Harris et al. 2022; Turner et al. 2022). ATP production and O_2_ consumption were normalized to wet tissue weight.

Hydrogen peroxide (H_2_O_2_) emission in permeabilized red GA fibers was measured with Amplex Red reagent as previously described (Anderson et al. 2007). Specific information can be found within the Data S8. Rates of H_2_O_2_ emission before and after the addition of succinate were calculated via the slope of ΔF/min after subtracting background and were normalized to wet fiber weight.

### 2.5 GSH/GSSG Measurement in Muscle

Colorimetric determination of total glutathione, reduced glutathione (GSH), oxidized glutathione (GSSG) and GSH/GSSG was assayed in a microplate reader (Biotek Epoch Microplate Spectrophotometer, Winooski, Vermont). For total glutathione, ~ 5 mg of frozen GA powder was homogenized in 150 μL of TEE buffer (10 mM Tris, 1 mM EDTA, 1 mM EGTA, 0.5% Tween‐20, pH 7.4). For GSSG assessment, samples followed the same procedure but included more tissue (~ 15 mg) and were homogenized in 0.3 mM Methyl‐2‐inylpyridinium (M_2_VP) in TEE buffer. Homogenates were centrifuged at 10,000 × *g* for 10 min, and pellets were discarded. Next, protein concentration was determined for each sample using the Pierce 660 nm Protein Assay. To fit within the standard curve, GSH samples were diluted 10‐fold. A consistent amount of protein/sample was added to each well in a volume of 25 μL. Standards of 0.05 μM, 0.125 μM, 0.25 μM, 0.75 μM, and 1.5 μM were run in triplicates with either TEE buffer (GSH) or TEE buffer + M_2_VP (GSSG). Glutathione Reductase (10 U/mL) mixed with DTNB (1 mM) (1:1 ratio), totaling 50 μL, was added to each well and incubated at room temperature for 10 min. Finally, 25 μL of NADPH was added to each well and the plate was immediately placed into the reader. The reaction was read at 405 nm every minute for 6 min. GSH was determined by subtracting GSSG from the total glutathione concentration.

### 2.6 Calcium Uptake in Isolated Mitochondria

Excised TA muscles were weighed and placed on ice‐cold mitochondria isolation medium (MIM) (300 mM Sucrose, 10 mM Na‐HEPES, 0.2 mM EDTA, 1 mM EGTA, pH 7.2 adjusted with glacial acetic acid). The tissue was finely minced and digested with 10 mL trypsin (125μg/mL in MIM) on ice for two minutes. Following the addition of 10 mL trypsin inhibitor (650μg/mL in MIM + BSA [1 mg/mL]), the tissue was centrifuged at 600× g for 10 min at 4°C. The supernatant was collected and subsequently centrifuged at 800× g for 10 min at 4°C. This supernatant was collected and centrifuged at 8000× g for 15 min at 4°C. This supernatant was discarded, and the mitochondrial pellet was cleaned via aspiration of contaminants. Another round of centrifugation was conducted, and the final mitochondrial pellet was resuspended in a small volume of MIM.

Calcium retention capacity (CRC) and maximal uptake speed in isolated mitochondria were measured using Calcium green‐5 N, as previously described (Murphy et al. 1996). Fluorescence was measured at 37°C in the BMG LABTECH (BMG Labtech, Cary, NC) microplate reader following 5 μL injection of 0.6 mM CaCl_2_ per well every 42 s. CRC was calculated based on the total amount of free Ca^2+^ ingestion until the fluorescent signal became saturated (i.e., opening of mitochondrial permeability transition pore [mPTP]). Maximal calcium uptake speed was quantified by the highest rate of free calcium uptake achieved during a single injection.

### 2.7 TEM Sample Preparation, Acquisition, and Analysis

Harvested GA muscles were immediately placed into cold saline and separated into red and white components. Red GAs were fixed in 2.5% glutaraldehyde and 1% paraformaldehyde (Boudina et al. 2007). Post‐fixation, embedding, sectioning, and imaging were conducted at the University of Iowa Microscopy Core Facility. Images at 2000 × and 8000 × magnifications for mitochondrial and MERC measurements, respectively, were obtained using a Hitachi HT7800 transmission electron microscope (Hitachi, Tokyo, Japan). Following the blinding of experimental groups, all morphological parameters were quantified using ImageJ as previously described (Lam et al. 2021). In short, area/perimeter‐based measurements (i.e., mitochondrion circumference, area and circularity and SR circumference and area) were assessed using the free hand tool to encircle all intermyofibrillar mitochondria. The line tool was used to measure the MERC length (i.e., lengths of individual SR membranes at the OMM) and MERC width (i.e., the closest distance between the cytosolic faces of the SR and OMM). Only ribosome‐free MERCs with a width within 50 nm were considered for measurement (Lu et al. 2022). Mitochondrial MERC coverage was quantified by determining the cumulative distance of MERC length(s) at an individual mitochondrion in relation to that same mitochondrion's circumference and expressed as a percentage. SR MERC coverage was measured as the percentage of an SR membrane's circumference in contact with the OMM.

### 2.8 Histological Analysis

For all histological analyses, dissected plantares were pinned and frozen in liquid nitrogen‐cooled isopentane. Prior to sectioning, frozen muscles were embedded in optimal cutting temperature compound. Cross‐sectional 5 μm serial sections were obtained using a Leica CM1520 cryostat (Leica Biosystems, Deer Park, IL). All slides were imaged on an Olympus BX63 microscope (Olympus, Tokyo, Japan). To assess fiber type and fiber diameter, sections were post‐fixed in acetone and blocked with 5% normal horse serum in 1× PBST. Next, slides were incubated in primary antibodies overnight at 4°C: anti‐MyHC 1/2A/2B (BA‐F8/SC71/BF‐F3 from Developmental Studies Hybridoma Bank, Iowa City, IA) and anti‐Laminin (L9393 from Sigma‐Aldrich). The following day, slides were incubated for 30 min in combined secondary antibodies: 647 nm IgG2B (A21242), 555 nm IgG1 (A21127), 488 nm IgM (A21042), and 405 nm IgG (A31556B). Three washes in 1× PBST preceded each of the steps. Finally, coverslips were mounted on each slide using Vectashield Plus Antifade Mounting Medium (H‐1900; Vector Labs, Newark, CA). Following the blinding of experimental groups, analysis of minimum Feret Diameter and fiber composition was performed on ImageJ software, as previously described (Turner et al. 2022). On average, 350 myofibers were inspected per section.

### 2.9 Isolation of Mitochondria‐Associated ER Membranes (MAMs)

Subcellular MAM fractions were obtained from the GA muscle using a previously described protocol in muscle (Lu et al. 2022; Wieckowski et al. 2009). Additional details can be found in Data S8. For every fractionation, both GAs were used. Protein concentration was determined using the Pierce 660‐nm Protein Assay.

### 2.10 Immunoblot Analysis

Whole GA lysates were prepared for SDS‐PAGE as previously described (Fuqua et al. 2019). Isolated MAM fractions were prepared for SDS‐PAGE by mixing the final samples (Please change to (suspended in mitochondrial resuspension buffer, details in Data S8) with 4X Loading Dye (50 mM Tris·HCl, pH 6.8, 1% sodium dodecyl sulfate (SDS), 10% glycerol, 20 mM dithiothreitol, 127 mM 2‐mercaptoethanol, and 0.01% bromophenol blue) in a 3:1 ratio and denatured at 95°C for 5 min. The primary antibodies used for this study were: ATP2A1/SERCA1 (Cell Signaling, 12293), VDAC1 (Cell Signaling, 4661), Alpha‐tubulin (Abcam, ab7291), PERK (Cell Signaling, 3192), Citrate Synthase (Sigma, sab2701077) ASCL4 (Santa Cruz, sc‐365,230), OXPHOS antibody cocktail (Abcam, ab110413), and COX IV (Abcam, ab33985). PVDF membranes were used for all immunoblot analysis, and images were taken in the Li‐Cor Odyssey CLX system (LI‐COR Biosciences, Lincoln, NE, USA). Protein signals were normalized to Ponceau staining.

### 2.11 TMT Sample Preparation

Data  S8 outlines sample clean‐up, digestion, and data acquisition. This was followed by LC/MS–MS to normalize samples based upon spectral counting (Erdjument‐Bromage et al. 2018; Poston et al. 2013; Rappsilber et al. 2007). TMT labeling was conducted as previously described (Zecha et al. 2019). Essentially, equivalent aliquots were reconstituted in a 17.5 μL of triethylammonium bicarbonate (TEAB). Samples were mixed 2:1 w/w with TMT‐10‐plex labeling reagent from anhydrous acetonitrile. Reactions continued for 1 h at room temperature and were then quenched with hydroxyl amine. Once barcoded, the samples were combined. The pooled sample was pre‐fractionated into 8 fractions and a pass‐through fraction that contained the hydrolyzed reagents and dimers using a Pierce High pH Reversed‐Phase Peptide Fractionation Kit (Thermo Scientific, 84,868) according to the manufacturer's instructions.

### 2.12 Bioinformatic Analyses

Several tools were used to characterize the proteome of isolated MAMs. MetaMass, a publicly available template for excel and R, comprises a comprehensive list of putative subcellular markers gathered from numerous publications and databases such as UniProt Knowledgebase (UniProtKB) and Human Protein Atlas (HPA) (Lund‐Johansen et al. 2016). Using this, we compared our proteome to others that included proteins only annotated to a single subcellular compartment. Overlap of proteins (or lack thereof) was detected using the conditional formatting function within Microsoft Excel. The clusterProfiler package in R was used to perform Gene Set Enrichment Analysis of altered GO terms in the two group comparisons (eAMD/HYA and eAMD+Ex/eAMD) (Wu et al. 2021). ClusterProfiler graphs were downloaded as scalable vector graphics (SVG) and visually adapted using the open‐source, vector graphics editor InkScape. All diagrams were created using BioRender Illustration Software.

### 2.13 Statistical Analysis

One‐way ANOVA analysis was used to compare HYA, eAMD, eAMD+Ex, or HYA, eAMD, and aAMD groups. The Kruskal–Wallis test was performed to analyze differences in the proteomes of HYA, eAMD, and eAMD+Ex groups. Based on several considerations collectively supported by Bantscheff et al., Ow et al., Savitski et al., and particularly Pascovici et al., *p* value corrections for multiple comparisons were not employed (Bantscheff et al. 2007; Ow et al. 2009; Pascovici et al. 2016; Savitski et al. 2011). The most important considerations were: (a) limited number of proteins identified in the muscle MERC fractions, which limits the ability to sort them into meaningful categories that may help describe functional phenotypic changes; (b) the quantitation at the MS/MS level using TMT labeling, which leads to ratio compression and smaller effect sizes and consequently higher *p* values, meaning that very few proteins (if any) might pass the corrections; and (c) our study design that juxtaposed the proteins inversely modulated by aging and exercise, adding further rigor to the identification and analysis of critical proteins and pathways contributing to the different phenotypes observed. Data are presented as mean ± SE. Violin plots are represented with first quartile, median, and third quartile. Proteomic comparisons were conducted using a Mann–Whitney *U* Test. Values of *p* < 0.05 were considered significant. All statistical tests were performed using GraphPad Prism software v. 10.3.1 (GraphPad Software, Boston, MA, USA).

## 3 Results

### 3.1 Regular Exercise Prevents Morphological and Functional Aspects of Early Dysfunction in Aging Muscle

**FIGURE 1:**
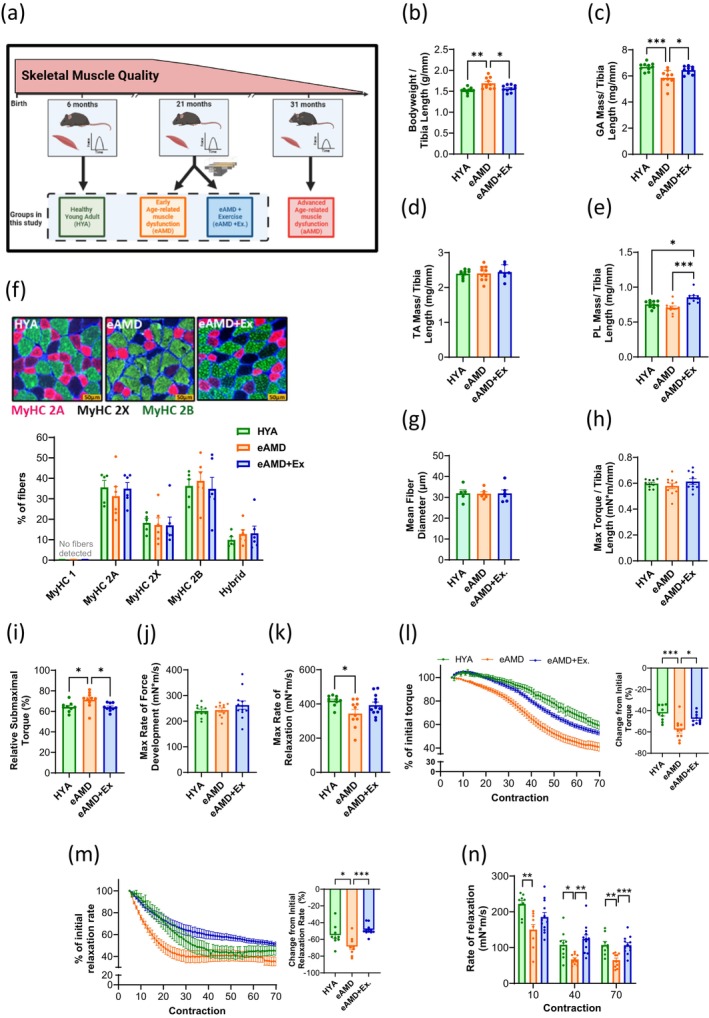
Effects of aging and exercise on skeletal muscle characteristics. (a) Diagram depicting skeletal muscle health across the lifespan and groups involved in the present study. (b) Bodyweight and wet muscle weight for (c) gastrocnemius, (d) tibialis anterior, and (e) plantaris normalized to tibia length (*n* = 10). (f) Top‐ Representative images of immunofluorescent (IF) staining of MyHC isoforms in each group. Bottom‐ Percentage of different MyHC isoforms in PL muscle fibers (*n* = 5–6). (g) Mean MinFeret diameter of PL fibers considering all fiber types (*n* = 5–6). (h) Maximal isometric torque (150 Hz) of plantar flexors normalized to tibia length (*n* = 10–12). (i) Submaximal isometric torque (50 Hz) expressed as a percentage of maximal torque (*n* = 9–10). (j) Maximal rate of force development during 150 Hz stimulation (*n* = 10–12). (k) Maximal rate of relaxation during 150 Hz stimulation (*n* = 10–12). (l) Left‐ torque values at each contraction averaged within groups during repetitive stimulation protocol. Right‐ percentage of initial torque lost after 70 contractions at 50 Hz (*n* = 9–12). (m) Left‐ rates of relaxation at each contraction averaged within groups during repetitive stimulation protocol. Right‐ percentage of initial relaxation rate after 70 contractions at 50 Hz (*n* = 9–12). (n) Individual values for rate of relaxation at different points during the repetitive stimulation protocol (*n* = 9–12). Data are means ± SEM; *p < 0.05, **p < 0.01, ***p < 0.001. HYA, healthy young adult; eAMD, early age‐related muscle dysfunction; eAMD+Ex, early age‐related muscle dysfunction following 6–8 weeks of regular endurance exercise.

To elucidate mechanisms preceding the onset of advanced age‐related muscle dysfunction (aAMD), we first compared animals of 6, 21 and 31 months of age. Compared to healthy young adults (HYA), animals at 31 months of age displayed significantly lower hindlimb muscle mass (i.e., of GA, PL and TA) and force (Figure S1A–E). The PL muscles of aAMD animals were the only to display the presence of Type I fibers and they also had preferential atrophy of Type 2B fibers compared to their younger counterparts, which is indicative of age‐related denervation/reinnervation (Figure S1F,H). On the other hand, both fiber type distribution and fiber diameter remained preserved in PL muscles of animals at 21 months of age (eAMD) (Figure S1F–H). Additionally, eAMD animals had unaltered TA and PL muscle masses, a modest decline in GA muscle mass, and preserved muscle force (Figure S1A–E). Based on these data, and a recently developed definition of sarcopenia in mice consistent with the European Working Group on Sarcopenia in Older People (EWGSOP) (i.e., low muscle strength, muscle mass and physical performance in relation to young adult counterparts) (Kerr et al. 2024), we considered this group to be at an early stage of age‐related muscle dysfunction (i.e., eAMD) (Figure 1a). Next, we investigated the impact of regular exercise during eAMD by subjecting a separate cohort of 19‐month‐old animals to 6–8 weeks of progressive treadmill training (i.e., finishing at 21‐months of age; eAMD+Ex group). This exercise regimen was sufficient to prevent bodyweight gain associated with sedentary aging, improve exercise capacity, and decrease fatigability (Figure 1b and Figure S2). Moreover, regular exercise effectively prevented age‐related atrophy in GA muscles, increased plantaris (PL) muscle mass, without altering TA muscle mass (Figure 1c–e). Notably, the exercise intervention did not alter fiber type or size in eAMD+Ex plantares (Figure 1f,g, Figure S3). Neither age nor exercise altered in vivo maximal torque (Figure 1h). However, at a submaximal stimulation frequency, eAMD animals were approximately 10% closer to attaining their maximal torque value(s) than HYA, and this was prevented by regular exercise (Figure 1i). Without changes to fiber composition, the attainment of a higher relative force at a submaximal stimulation frequency may result from inefficient contraction due to slower ECR cycles (Mayfield et al. 2023). Accordingly, eAMD animals did not present deficits in the rate of force development but exhibited reductions in maximum rate of relaxation compared to HYA (Figure 1j,k). Exercise also reverted this outcome (Figure 1j,k). eAMD animals were more prone to peripheral fatigue than young and exercise‐trained animals, as indicated by a larger decrease in force (58% vs. 43% and 48%, respectively) during repeated submaximal contractions (Figure 1l). As expected, all groups presented a progressive deterioration in the rate of relaxation during the fatigue protocol, but such decline was more pronounced in eAMD animals (Figure 1m). Moreover, eAMD animals displayed impaired rates of relaxation throughout the entirety of this assessment, suggesting that relaxation is impaired at all levels of fatigue (Figure 1n). Collectively, these data identify the slowing of muscle relaxation as a critical functional deficit occurring early in the development of age‐related muscle dysfunction, and that regular endurance exercise prevents this outcome.

### 3.2 Exercise Prevents Early Age‐Related Changes in Mitochondrial Ultrastructure and ROS Emission

**FIGURE 2:**
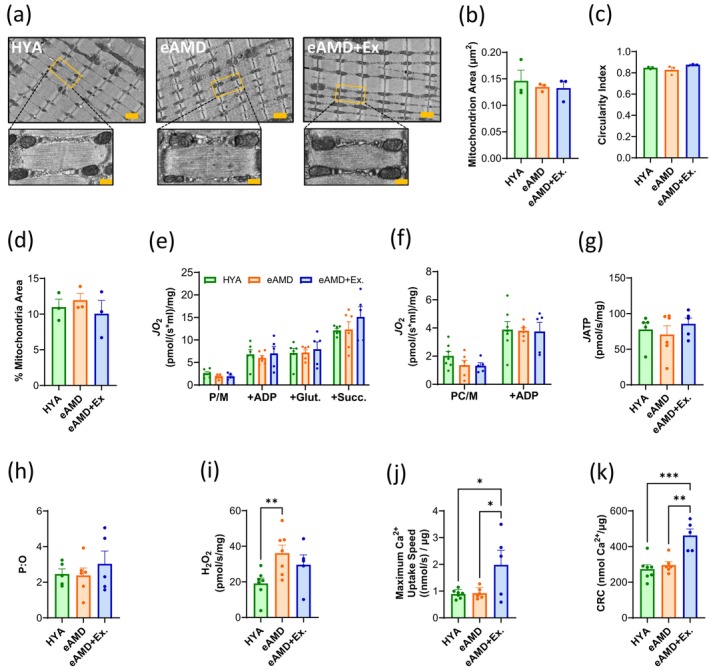
Effects of aging and exercise on skeletal muscle mitochondria. (a) Representative TEM images of intermyofibrillar (IM) mitochondria in red gastrocnemius from each group (*n* = 3 animals, 3–4 images per animal, which also applies to (b–d)). Yellow scale bars represent 1 μm and 250 nm in the larger and smaller images, respectively (b) Mean surface area of individual IM mitochondria. (c) Mean circularity index (Y = 4π*[SA/(perimeter2)] of individual IM mitochondria. (d) Percentage of quantified area occupied by IM mitochondria. (e) High‐resolution respirometry measurements of oxygen flux (*J*O_2_) using carbohydrate‐based and (f) fatty acid‐based substrates in permeabilized red gastrocnemius fibers (*n* = 5–7). (g) ATP production rate (*J*ATP) in permeabilized fibers after injection of ADP; derived via NADPH autofluorescence (*n* = 5–7). (h) Total ATP production from (g) normalized to the amount of oxygen consumed in the same amount of time. (i) Hydrogen peroxide (H_2_O_2_) emission following injection of succinate (no ADP) in permeabilized red gastrocnemius fibers (*n* = 5–7). (j) Highest rate of Ca^2+^ uptake achieved during a single Ca^2+^ pulse in isolated TA mitochondria (*n* = 5–7). (k) Total Ca^2+^ retention capacity (CRC) prior to swelling and mitochondrial permeability pore (mPTP) opening in isolated TA mitochondria (*n* = 5–7). P/M‐ Pyruvate/Malate; Glut‑Glutamate; Succ‐ Succinate; PC/M‐ Palmitoylcarnitine/Malate. Data are means ± SEM. Bar graphs are means ± SEM; *p < 0.05, **p < 0.01, ***p < 0.001. HYA, healthy young adult; eAMD, early age‐related muscle dysfunction; eAMD+Ex, early age‐related muscle dysfunction following 6–8 weeks of regular endurance exercise.

The observed deficits in muscle relaxation suggested that bioenergetics may be impaired early with aging. Therefore, we next assessed mitochondrial ultrastructure in red gastrocnemius (GA) fibers using transmission electron microscopy (TEM) imaging to identify potential intrinsic mitochondrial deficits in eAMD. We observed no differences in surface area or circularity of individual intermyofibrillar (IM) mitochondria across the three groups (Figure 2a–c). Additionally, relative mitochondrial area in TEM images remained unchanged and the expression of electron transport chain (ETC) subunits was not consistently higher in eAMD+Ex vs. eAMD or HYA groups (Figure 2d, Figure S4A).

We then interrogated mitochondrial respiratory function in saponin‐permeabilized red gastrocnemius (GA) fibers. Contrary to expectation, maximal rates of respiration (*J*O_2_) utilizing either carbohydrate‐based or fatty acid‐based substrates were not different across groups (Figure 2e,f). The efficiency of ATP synthesis, measured by the P:O ratio and rate of ATP production (*J*ATP), was also not affected in early age‐related muscle dysfunction or by regular exercise (Figure 2g,h). However, fibers from eAMD muscles exhibited higher levels of H_2_O_2_ emission compared to HYA, and this was mitigated by exercise (Figure 2i). Even though relative GSH:GSSG ratios did not reach statistical significance across groups, eAMD muscles had a ~ 50% lower ratio than the other groups, suggesting a potential increase in oxidant load (Figure S4B–D).

Because mitochondrial Ca^2+^ uptake directly regulates various TCA cycle enzymes and ROS production, we then evaluated Ca^2+^ uptake by isolated mitochondria from the tibialis anterior (TA) muscle. Neither maximal Ca^2+^ uptake rate nor Ca^2+^ retention capacity was altered in eAMD (Figure 2j,k). However, exercise significantly increased both measures of mitochondrial calcium handling (Figure 2j,k). Overall, these results highlight increased ROS emission as a potential driver of the phenotype in eAMD despite preserved overall mitochondrial content, Ca^2+^ uptake dynamics, and maximal respiratory capacity. Importantly, regular exercise effectively prevented this early aging‐related change.

### 3.3 MERC Ultrastructure Is Altered With Age and Normalized by Exercise

**FIGURE 3:**
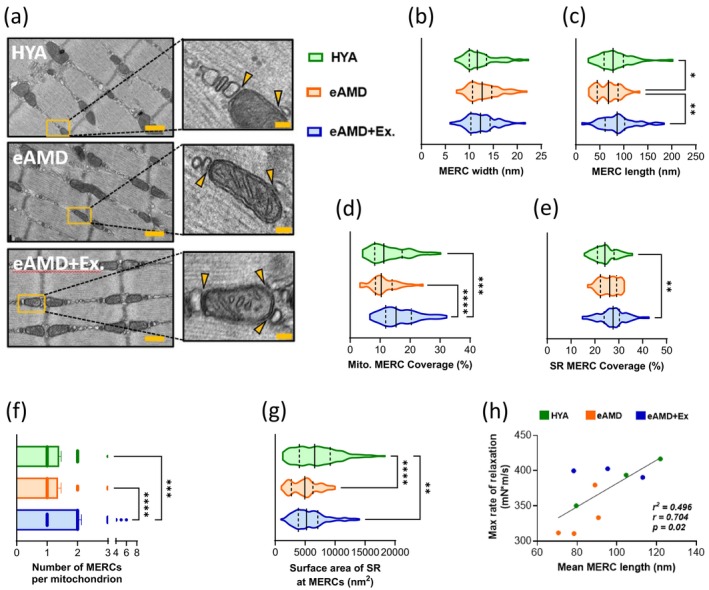
Impact of aging and exercise on skeletal muscle MERC ultrastructure. (a) Representative TEM images for quantification of MERCs in red gastrocnemius. Yellow arrows indicate MERC region of interest (i.e., physical contact between mitochondria [gray] and SR [white]) (*n* = 3 animals/group, 2–3 images per animal, 5–10 sites per image, which also applies to (b–h)). Yellow scale bars represent 500 nm and 50 nm in the larger and smaller images, respectively. (b) Distance between the cytosolic face of sarcoplasmic reticulum (SR) and the outer mitochondrial membrane (OMM). (c) Length of individual SR membranes in contact with OMM. (d) Relative percentage of mitochondrion perimeter covered by SR membrane contact(s). (e) Relative percentage of individual SR membrane perimeter facing the OMM at MERCs. (f) Number of ER/SR contact sites per mitochondrion (MERCs) in each group. Violin plots display (g) Surface area of individual SR membranes at MERCs. (h) Pearson correlation coefficient (*r*) and coefficient of determination (*r* ^*2*^) resulting from Mean MERC length and Maximal rate of relaxation across HYA, eAMD and eAMD+Ex groups. Violin plots display 1st quartile, median, and 3rd quartile. *p < 0.05, **p < 0.01, ***p < 0.001., ****p < 0.0001. HYA, healthy young adult; eAMD, early age‐related muscle dysfunction; eAMD+Ex, early age‐related muscle dysfunction following 6–8 weeks of regular endurance exercise.

Since mitochondrial respiratory function was preserved in early age‐related muscle dysfunction and unaffected by exercise, we then hypothesized that MERC ultrastructure would be altered at that stage potentially contributing to slower relaxation rates by compromising the communication between mitochondria and the SR. High‐magnification TEM images (8000×) of the red portion of GAs were captured to assess MERC ultrastructure using previously described parameters (Figure 3a) (Giacomello and Pellegrini 2016). MERC width describes the cytosolic cleft distance between the SR membrane and the mitochondrial outer membrane. Because many of the molecules exchanged at MERCs are subject to the diffusion laws of Einstein and Fick, a larger separation (i.e., increased width) between mitochondria and SR would decrease the efficiency of nucleotide (e.g., ATP) and/or ion (e.g., Ca^2+^) transport across organelles (Giacomello and Pellegrini 2016). On the other hand, MERC length defines the stretch of individual SR membrane segments parallel to the outer mitochondrial membrane at contact sites. Compared to HYA, MERC width was not altered in eAMD or eAMD+Ex MERCs. This suggests that exchange efficiency was preserved at this early stage of muscle dysfunction (Figure 3b). However, MERC length was significantly lower with aging, and this was prevented with regular exercise (Figure 3c). Assuming that MERC length is proportional to the cumulative transport potential of molecules across the two organelles, MERCs in eAMD may have a diminished capacity for exchanging nucleotides and ions between the SR and mitochondria potentially impacting contractile function. Along with the length of individual MERC sites, we also assessed MERC coverage of the two organelles. Mitochondrial MERC coverage describes the cumulative MERC lengths relative to the respective mitochondrion perimeter and SR MERC coverage describes the SR length at a MERC relative to its perimeter. MERC coverage of both organelles was unaltered in early aging but was increased with regular exercise to levels higher than healthy young adults (Figure 3d,e). This is likely due to an increase in number and not size of SR moieties present at the MERCs following exercise training (Figure 3f,g). Of note, MERC length was strongly associated with the in vivo rate of muscle relaxation across groups, with the former explaining ~ 50% of the variation of the latter (Figure 3h). Collectively, these data suggest that MERC ultrastructure is altered in early AMD and that the exercise‐mediated modulation of MERC ultrastructure is beneficial to muscle function.

### 3.4 MAM Protein Composition Is Differently Impacted in Early Aging and by Regular Exercise

**FIGURE 4:**
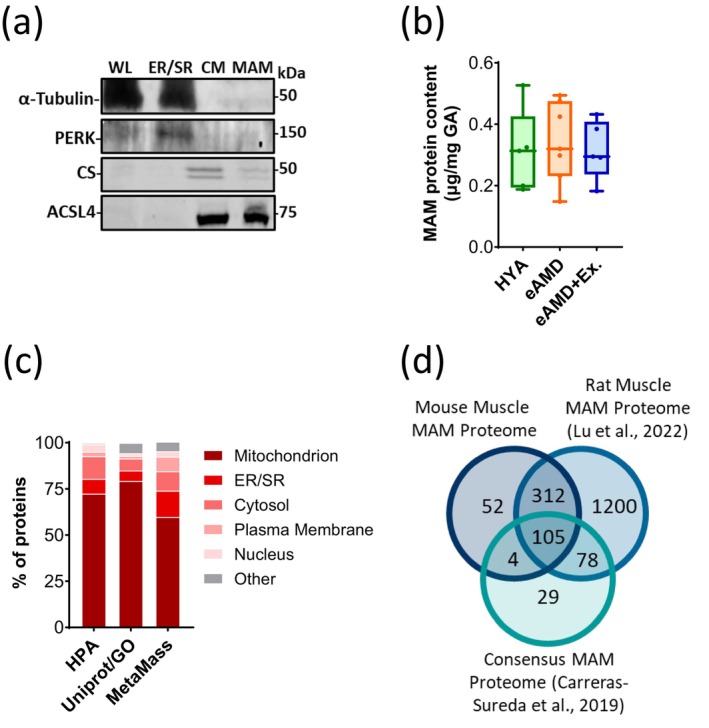
Characterization of skeletal muscle MAM proteome. (a) Representative immunoblots of muscle tissue denoting the obtained fractions of whole lysate (WL), endoplasmic and sarcoplasmic reticulum (ER/SR), crude mitochondria (CM) and MAMs. (b) Total protein yield of MAM isolations normalized to amount of GA tissue used (*n* = 5–7). (c) Distribution of proteins with single annotation in the MAM proteome according to HPA, Uniprot/GO and MetaMass. (d) Comparisons between mouse skeletal muscle MAM proteome (current study), rat skeletal muscle MAM proteome (Lu et al. 2022), and consensus MAM proteins (Carreras‐Sureda et al. 2019). HYA, healthy young adult; eAMD, early age‐related muscle dysfunction; eAMD+Ex, early age‐related muscle dysfunction following 6–8 weeks of regular endurance exercise.

Since MERC ultrastructure was altered with aging and exercise, we next interrogated if changes in proteins expressed at MERCs would be associated with early age‐related muscle dysfunction and the benefits of exercise. To address this question, we first isolated mitochondria‐associated endoplasmic reticulum membranes (MAMs) following a previously established protocol (Wieckowski et al. 2009) with a few modifications (see Materials and Methods section and Data S8), and then probed equal amounts of fractionated proteins for markers of specific cellular compartments. The cytosolic protein α‐tubulin was present in the whole‐tissue lysate and in the ER/SR fraction. Conversely, the residing ER/SR protein Eukaryotic Translation Initiation Factor 2 Alpha Kinase 3 (EIF2K3, also referred to as PERK) was enriched in the ER/SR fraction. Both α‐tubulin and PERK were undetectable in the crude mitochondria and MAM fractions. In this protocol, MAM fractions are obtained from the crude mitochondria fraction. Therefore, while MAM fractions contained only traces of the mitochondrial matrix protein Citrate Synthase (CS), both fractions were enriched with the canonical MAM marker, Acyl‐CoA Synthetase Long Chain Family Member 4 (ACSL4) (Wieckowski et al. 2009) (Figure 4a). Of note, total protein content in MAM fractions relative to the amount of GA tissue processed did not differ across groups indicating that overall quantitative changes in the MAM proteome are not present at this early stage of aging (Figure 4b). Next, we TMT‐labeled MAM fractions from the three groups and subsequently submitted those samples through LC–MS identifying a total of 473 proteins. To gain insight into the cellular locations of these proteins, we indexed our MAM proteome across the Human Protein Atlas (HPA), UnitProt/GO using MetaMass (Lund‐Johansen et al. 2016). Accordingly, ~ 75% of the proteins in these fractions were located at either the mitochondrion or ER/SR. The next most abundant cellular compartment for the identified proteins was the cytosol. This was not unexpected as some MERC proteins do not physically interact with the ER/SR or the outer mitochondrial membrane but are located between these organelles (Zhou et al. 2023) (Figure 4c). Subsequently, we juxtaposed our proteome to previous investigations of MAM fractions. Over 85% of identified proteins (i.e., 417 proteins) aligned with work recently performed in rat skeletal muscle (Lu et al. 2022), indicating that the protein composition of skeletal muscle MAMs is well‐conserved in rodents (Data S1). Among those, 105 proteins aligned with previously established consensus MAM proteins, which were based on studies of MERCs across a myriad of non‐contractile cells and tissues (Carreras‐Sureda et al. 2019). The remaining group of 312 MAM proteins appears unique to skeletal muscle given their presence in muscle MAM fractions obtained from mice (current work) and rats (Lu et al. 2022) (Figure 4d).

**FIGURE 5:**
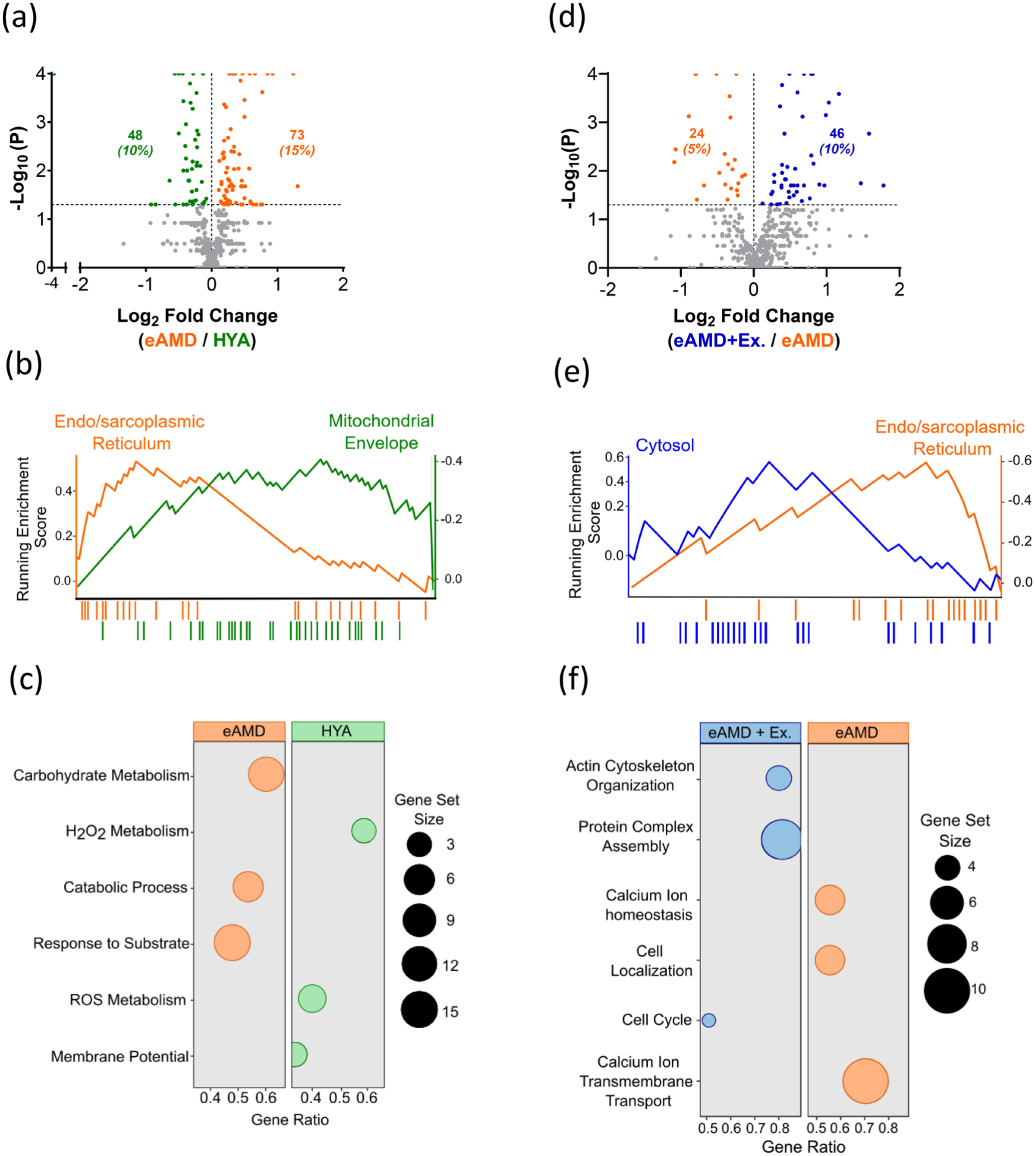
Effects of aging with or without regular exercise on skeletal muscle MAM proteome. (a) Volcano plot comparing the MAM proteome of eAMD vs. HYA (*n* = 3). Number and percentage of differentially expressed proteins (colored) relative to the entire proteome (473 proteins) are shown. Horizontal dashed line denotes the lower limit for significance (i.e., −log_10_(*p*) > 1.3). (b) Top‐enriched (*p* < 0.05) discrete organellar proteins (genesets) from GO:CC (GSE analysis) of differentially expressed genes or proteins (DEGs) from eAMD vs. HYA comparison in ClusterProfiler R package (Outcome was *N* = 25 and 33 total proteins for endo/sarcoplasmic reticulum and mitochondrial envelope, respectively). (c) Top‐enriched genesets (*p* < 0.05) from GO:BP (GSE analysis) in ClusterProfiler R package. (d) Volcano plot comparing proteome of eAMD+Ex vs. eAMD MAMs (*n* = 3). (e) Top enriched (*p* < 0.05) discrete organellar proteins (genesets) from GO:CC of DEGs from eAMD+Ex vs. eAMD comparison in ClusterProfiler R package (outcome was N = 17 and 25 total proteins for endo/sarcoplasmic reticulum and cytosol, respectively). (f) Top enriched genesets (*p* < 0.05) from GO:BP (GSE analysis) in ClusterProfiler R package for eAMD+Ex vs. eAMD.

To interrogate the potential effects of early biological aging on skeletal muscle MAM protein composition, we then compared the subcellular proteomes of HYA and eAMD animals. Out of the 473 MAM proteins identified, 121 changed with age. From those, 73 proteins (15%) were increased, and 48 proteins (10%) decreased in eAMD (Figure 5a, Data S2). Due to the dynamic localization of proteins at MAMs, we sought to characterize which subcellular locations were most affected. To this end, we analyzed the 121 significantly altered MAM proteins using Gene Set Enrichment Analysis (GSEA) via Gene Ontology. In terms of cellular compartments (i.e., GO:CC), eAMD MAMs displayed an increased enrichment of SR/ER‐related proteins, while HYA MAMs were mostly enriched with Mitochondrial Envelope proteins (Figure 5b, Data S3). Related to biological processes (i.e., GO:BP), proteins involved in the removal of ROS (e.g., cytochrome c (Cycs), superoxide dismutase 2 (SOD2), and nicotinamide nucleotide transhydrogenase (Nnt)) and regulation of membrane potential were enriched in HYA MAMs, while eAMD MAM fractions displayed enrichment of proteins responsive to glycolytic and catabolic substrates (Figure 5c; Data S4).

To examine if changes in the MAM proteome underlie the beneficial adaptations of muscle to regular exercise, we compared eAMD and eAMD+Ex groups. Of the 473 proteins identified, 70 significantly changed with exercise. Of those, 46 proteins (10%) were increased in eAMD+Ex, while 24 proteins (5%) were decreased (Figure 5d, Data S2). In terms of cellular compartments, eAMD MAMs again displayed enrichment of ER/SR‐related proteins when compared to eAMD+Ex. Interestingly, the top‐enriched discrete cellular compartment for proteins changing with exercise was the cytosol, suggesting exercise‐specific adaptations to MAMs (Figure 5e, Data S5). The biological processes mostly affected by regular exercise comprised broad categories, including cytosolic modifications (e.g., actin cytoskeleton organization and protein complex assembly) and cell cycle (Figure 5f). Comparatively, the pool of MAM proteins enriched in aging sedentary muscle suggested an increased reliance on ER/SR‐regulated processes related to calcium homeostasis (e.g., calcium ion homeostasis and transmembrane transport) (Figure 5f, Data S6). Altogether, these data support a paradigm in which exercise not only prevents changes in ER/SR proteins at MAMs observed early in sedentary aging but also promotes the enrichment of cytosolic proteins at MAMs likely contributing to improvements in ER/SR–mitochondria crosstalk.

### 3.5 Aging and Exercise Inversely Modulate the Expression of Several MAM Proteins

**FIGURE 6:**
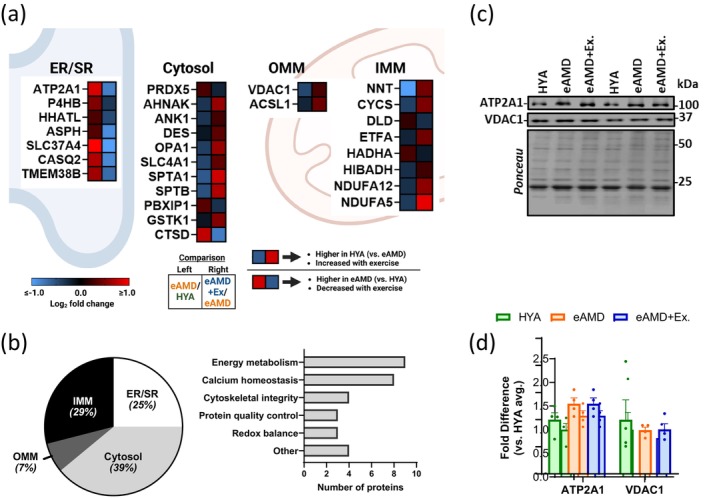
MAM proteins inversely modulated in aging groups that were either sedentary or exercised regularly. (a) Inversely regulated MAM proteins (with age and exercise) according to their location at MERCs. Heatmaps represent respective quantitative proteomic comparisons (refer to keys at the bottom). (b) Distribution of inversely modulated MAM proteins across MERC subcellular locations (left) and molecular functions (right). These were collectively curated from NCBI and GeneCards. (c) Representative immunoblots and (d) Quantification of ATP2A1 and VDAC1 expression in whole GA lysates (*n* = 4). HYA, healthy young adult; eAMD, early age‐related muscle dysfunction; eAMD+Ex, early age‐related muscle dysfunction following 6–8 weeks of regular endurance exercise.

To identify MAM proteins most relevant to muscle health during aging, we collectively examined proteins that were inversely modulated by sedentary aging and exercise. In total, 28 proteins fell into this category. Of those, 17 were higher, while 11 were lower in HYA and eAMD+EX vs. eAMD (Figure 6a). Interestingly, all proteins present at higher levels in MAMs of young adult and exercised animals were located in either the mitochondrion or the cytosol and were generally involved in energetics and cytoskeleton integrity. On the other hand, most of the proteins present at higher levels in MAMs of animals with early age‐related muscle dysfunction were located in the ER/SR and were generally involved in protein quality control and calcium dynamics (Figure 6a,b, Data S7). We next tried to obtain insights into whether those changes were secondary to the expression of these proteins in the whole muscle tissue. To address this, we assessed the expression of MAM proteins found at the SR and at the mitochondrial outer membrane (i.e., ATP2A1 and VDAC1, respectively) in whole GA muscle lysates. Their whole‐tissue expression was unaltered with sedentary aging or with exercise, despite their either increased (i.e., ATP2A1) or decreased presence at MAMs (i.e., VDAC1) of eAMD vs. HYA and eAMD+Ex (Figure 6a,c,d). Collectively, these observations suggest that at least some of the MERC protein changes occurring in early age‐related muscle dysfunction result from their altered localization at the contact sites and that regular exercise can prevent this.

## 4 Discussion

Due to their terminal differentiation, skeletal muscle fibers are particularly susceptible to biological aging. After age 50 in humans, muscle mass decreases at ~ 1%–2% per year and this rate nearly doubles after 60 (von Haehling et al. 2010). Occurring in parallel is a decrease in force output that reduces physical function, compromises independence, and increases the risk of mortality (Lang et al. 2010). Therefore, the most effective treatments for sarcopenia are likely those that are targeted at the earliest stages of aging. Regular exercise is currently the only effective therapy for age‐related muscle dysfunction. However, many social, economic, and health‐related barriers exist that prevent individuals from maintaining consistent activity. Despite our considerable understanding of the maladaptive processes transpiring at advanced stages of muscle aging, our knowledge of the initial molecular events leading to sarcopenia remains elusive. A better understanding of this is therefore required to begin developing effective therapies. Based on a recently adapted definition from EWGSOP for sarcopenia in mice, we studied a group of animals considered to be at an “early” stage in the progression of sarcopenia or age‐related muscle dysfunction (eAMD) (Kerr et al. 2024). These mice presented either mild or no muscle atrophy with preserved maximal force. Despite this, we show that relaxation was much slower in these mice in both fatigued and non‐fatigued conditions. As a result, their ECR cycles were elongated, creating premature tetanic fusion and higher force –relative to their max– during submaximal contractions. Though this may be partially beneficial for a few successive muscle contractions, we observed that it may predispose the muscles to fatigue over a longer period of time. This is supported by recent data in humans that showed an association between longer relaxation time and perceived fatigue (Mast et al. 2024). Unexpectedly, we observed force potentiation in the HYA and eAMD+Ex groups during the fatigue protocol. We believe this could be due to greater activation of myosin regulatory light chain by myosin light chain kinase, which has been previously shown to play a role in the potentiation of force during repetitive stimulations (Ryder et al. 2007). However, further studies are required to delineate whether this is the case. Since their fiber type distribution was unaltered, these functional changes took place before age‐related denervation and subsequent type 2 to type 1 fiber switching. Of note, because our functional measurements involve direct stimulation of the tibial nerve, and because motor neurons conduct electrical signals (i.e., action potentials) ~ 100 times faster than muscle fibers, we can rule out neuronal‐mediated deficits as a cause of reduced muscle relaxation (Loeb and Ghez 2000; Zollinger et al. 2015). Collectively, these observations indicate that the slowing of muscle relaxation is likely the first functional impairment to occur with aging in males, and that its driving mechanisms reside within the myofibers. Whether this is also true in females will require further investigation.

To begin interrogating potential causes of this, we studied an additional group of eAMD mice that regularly exercised. Our 8‐week intervention was effective at preventing the decline of relaxation rate while also mitigating muscle fatigue. Due to the heavy reliance of these contractile functions on a constant supply of ATP, we turned our attention to muscle mitochondria. In fact, dysfunction of mitochondria occurs ubiquitously in more advanced stages of aging (Marzetti et al. 2024). However, during early AMD we did not observe any differences in mitochondrial morphology or content. Moreover, maximal respiratory capacity was largely unaffected by aging and exercise. Taken together, this suggests that intrinsic mitochondrial properties remain mostly preserved at early stages of sarcopenia. Mitochondrial H_2_O_2_ emission, however, was increased with aging and attenuated by exercise. These findings implicate elevated mitochondrial ROS emission as an important (mal)adaptation in muscle that precedes the major contractile deficits normally observed during advanced aging. Because the oxidation of macromolecules has been shown to compromise muscle function in different settings, future studies are required to clarify (a) which specific molecules may be modified by mitochondrial ROS emission and (b) whether these are related to alterations in muscle relaxation (Andersson et al. 2011; Powers et al. 2011).

Within muscle fibers, Ca^2+^ is not only critical for contractile function, but it also stimulates oxidative phosphorylation when imported into mitochondria (Duchen 2000). In that sense, an impaired ability of mitochondria to uptake Ca^2+^ could limit the acceleration of ATP resynthesis required for SERCA‐mediated SR Ca^2+^ reuptake, thus delaying muscle relaxation. However, our results demonstrated that when exposed to excess Ca^2+^, neither speed of mitochondria Ca^2+^ uptake nor total retention capacity were impacted in early aging muscle. Though exercise improved these to levels above HYA animals, the previous observation suggests that the intrinsic capacity of mitochondria to import Ca^2+^ does not contribute to the slowing of relaxation seen in eAMD. Next, we asked whether external mitochondrial interactions could be responsible for the phenotype observed in early aging. Indeed, such interactions are becoming increasingly recognized as critical regulators of intra and inter‐organelle homeostasis (Elbaz‐Alon 2017). In skeletal muscle, intermyofibrillar MERCs are particularly important as they represent physical interactions between the critical site of Ca^2+^ handling and regulation of contraction (i.e., the ER/SR) and ATP resynthesis (i.e., mitochondria). Therefore, we first examined these MERCs using well‐established morphological parameters: width, length, and coverage (Giacomello and Pellegrini 2016). According to laws of diffusion, MERC width is thought to alter the exchange efficiency of metabolites across organelles (Giacomello and Pellegrini 2016). Our findings indicated that MERC exchange efficiency was unaltered in eAMD and regular exercise groups, and likely did not contribute to the contrasting muscle phenotypes observed in these conditions. Interestingly, recent studies suggest that MERC clefts become wider in advanced sarcopenia, which may contribute to the exacerbated contractile dysfunction present at that timepoint (Lu et al. 2022). MERC length was lower in eAMD mice and restored with exercise. Assuming there is a relation between MERC length and the number of structures capable of ion/molecule exchange, the former should define the exchange capacity of an individual contact site. Because Ca^2+^ released from the SR can stimulate mitochondrial metabolism, and ATP exported from mitochondria is important for optimal SR ATPase (i.e., SERCA) function, a decreased capacity to exchange ions and nucleotides between the two organelles could directly lead to reductions in the rate of muscle relaxation. In fact, MERC length was able to explain ~ 50% of relaxation rate across HYA, eAMD, and eAMD+Ex groups (Figure 3h). Due to the limited sample size, however, we recognize that these data are preliminary and that investigations on Ca^2+^ exchange flux between the ER/SR and mitochondria should be conducted in future studies. Interestingly, exercise led to higher MERC coverage and number of contact sites than both eAMD and HYA (Figure 3d–f). Since the perimeter of SR moieties at MERCs was not altered by exercise, these observations suggest that exercise‐mediated MERC adaptations may be in part due to increased ER/SR structures (perhaps secondary to ER/SR biogenesis or reorganization) around mitochondria (Figure 3g). Overall, these observations highlight the potential impact of MERC ultrastructure on muscle contractile function. It is important to note that future studies using 3D reconstructions of muscle MERCs are required to reveal additional nuances related to changes at the contact sites during aging and/or exercise.

In order to execute their numerous cellular processes, MERCs rely on an eclectic assortment of localized proteins. Related to ECR cycles in muscle, it is likely that some of these are either directly (e.g., exchange of ions and nucleotides) or indirectly (e.g., redox balance) involved. We therefore sought to profile the MERC proteome across our three groups by isolating MAM‐enriched fractions from muscle. Total MAM protein concentration, which was shown to decrease at more advanced stages of aging, did not differ across groups (Lu et al. 2022). Next, LC/MS of the TMT‐labeled MAM fractions allowed us to determine both quantitative and qualitative changes in the proteome. As expected, 80%–90% of the 473 discovered proteins were annotated to either the mitochondrion, ER/SR or the cytosol (Figure 4c). Proteins linked to ER/SR lumen, mitochondrial matrix, nuclear, and plasma membrane were also present in the fractions. This could be at least partly due to physical interactions between these proteins and MERCs in vivo.

We observed that during early age‐related muscle dysfunction, 25% of MAM proteins were altered, with 15% being upregulated and 10% downregulated compared to healthy young adult MAMs (Figure 5a). Regular exercise in the older animals changed 15% of MAM proteins, upregulating 10% and downregulating 5% (Figure 5d). GSEA of eAMD vs. HYA animals revealed that aging led to a greater enrichment of ER/SR‐related proteins at MAMs (Figure 5b). On the other hand, HYA MAMs were enriched with mitochondrial proteins. This was an interesting finding, since “ER‐predominant” MAMs have been reported at more advanced stages of sarcopenia and may be attributed to decreased muscle oxidative capacity (Lu et al. 2022; Migliavacca et al. 2019). Further analysis of GO:BP showed that whereas ER/SR‐predominant MAM proteins are involved in catabolic and/or glycolytic processes, mitochondria‐predominant MAMs (i.e., HYA MAMs) primarily modulate redox‐related processes (Figure 5c). Thus, the latter may be partially responsible for the differences in H_2_O_2_ emission we observed. Additional studies on muscle mitochondria energetics and redox balance, such as in vivo P‐MRS, may provide additional important insights into this paradigm.

Compared to exercise‐trained older animals, sedentary animals maintained a greater enrichment of ER/SR‐related proteins; further implicating ER/SR‐predominant MAMs in muscle dysfunction (Figure 5e). Specifically, these MAMs showed higher levels of proteins involved in Ca^2+^ homeostasis (e.g., ATP2A1/SERCA1, RYR1, and TRDN) (Data S5). This may be due to compensatory upregulations of these proteins in response to diminished Ca^2+^ release unit coupling that has been shown to occur with sedentary aging (Michelucci et al. 2021). We hypothesized that exercise would effectively revert ER/SR‐predominant MAMs to the mitochondria‐predominant MAMs observed in HYA animals. Interestingly, MAMs from the eAMD+Ex. group instead displayed enrichment of cytosolic components (Figure 5e). Albeit some of the protein sets that were modified with exercise included mitochondrial proteins (e.g., NDUFA5/6/12), the majority were deputed to sarcoplasmic reorganization (e.g., SPTA1, SPTB, FLNC) (Data S5). In the context of MERCs, these may be relevant in restoring MERC stability and facilitating more efficient MERC morphology (e.g., greater MERC length).

Finally, we sought to identify proteins inversely modulated by aging and exercise. In this way, we could profile a specific subpopulation of MAM proteins more determinant to muscle health. We identified 28 candidate proteins that fit this description. These included seven localized to the SR, eleven to the cytosol, two in the outer mitochondrial membrane (OMM), and eight in the inner mitochondrial membrane (Figure 6a). Further inspection of whole‐tissue levels of markers for MERC sub‐compartments (e.g., ATP2A1/SERCA1‐ SR, VDAC1‐OMM) revealed that their overall expression was not changing in muscle (Figure 6c,d). Taken together with changes in the MAM proteome, this indicates that in HYA and eAMD+Ex., a larger proportion of VDAC1 and a lower proportion of ATP2A1 are localized to MERCs. These observations indicate that the localization of certain proteins to MERCs is also modulated. Indeed, increased density of VDAC proteins to MERCs seems to allow microdomains of rapid Ca^2+^ transfer from the ER/SR to mitochondria, impacting the overall exchange between organelles (Rosencrans et al. 2021).

In conclusion, the present study provides foundational information regarding the impact of aging and regular exercise on skeletal muscle health, MERC ultrastructure, and protein composition. Our findings denote preserved maximal torque, decreased relaxation rate, and increased fatigability as features of early age‐related muscle dysfunction that are prevented by regular aerobic exercise initiated at middle age. Further, our data implicate mitochondrial ROS emission and exonerate mitochondrial respiratory capacity as a main subcellular culprit. For the first time, we reveal that the MAM ultrastructure and proteome are inversely modulated by aging and exercise. The identification of inversely regulated MAM proteins in early age‐related muscle dysfunction and exercise can be used to generate hypotheses investigating the influence of the contact sites and their protein composition on muscle dysfunction. Future studies focusing on the regulation and function of these proteins should provide new and important insights into the mechanisms modulating skeletal muscle health. Lastly, investigation of these phenomena in different exercise modalities (e.g., resistance training) and females will be required to fully uncover the therapeutic relevance of these findings in the context of aging.

## Author Contributions

V.A.L. and R.O.P. conceived the original idea for this study. R.J.A., A.K., Q.S., M.P., E.C.‐M., and W.S. performed the experiments. L.‐S.S., E.J.A., and R.O.P. provided equipment and guidance for experiments. R.J.A. analyzed the data, and R.J.A., A.K., W.S., and V.A.L. interpreted the data. R.J.A. and V.A.L. wrote the manuscript. Q.S., M.P., L.‐S.S., E.J.A., and R.O.P. reviewed and revised the manuscript prior to submission.

## Conflicts of Interest

The authors declare no conflicts of interest.

## Supporting information


**Figure S1.** Phenotypic effects of aging in skeletal muscle. Refer to schematic in Figure 1A. (A) Bodyweight and wet muscle weight for (B) gastrocnemius, (C) tibialis anterior, and (D) plantaris muscles normalized to tibia length. (E) Maximal isometric torque of plantar flexors via stimulation (150 Hz) of the tibial nerve normalized to tibia length. (*n* = 4–12). (F) Percentage of different MyHC isoforms in PL muscle fibers (*n* = 3–6). (G) Mean MinFeret diameter of PL fibers considering all fiber types. (H) Mean MinFeret diameter of PL fibers separated by fiber type. Data are means ± SEM; *p < 0.05, **p < 0.01, ***p < 0.001, ****p < 0.0001. eAMD, early age‐related muscle dysfunction; eAMD+Ex, early age‐related muscle dysfunction following 6–8 weeks of regular endurance exercise; HYA, healthy young adult.
**Figure S2**. Effects of regular endurance exercise on 21‐month‐old mice (eAMD+Ex). (A) Bodyweight before and after 6‐to‐8‐week treadmill intervention (*n* = 12). (B) Total running distance during treadmill exhaustion test (*n* = 12). (C) Percentage of initial force lost after 70 repetitive submaximal (50 Hz) stimulations (*n* = 12). Pre—before exercise regular exercise, Post—after regular exercise. Data are individual values; **p < 0.01, ****p < 0.0001.
**Figure S3**. Effects of aging and exercise on plantaris fibers. (A) Mean MinFeret diameter of PL fibers separated by fiber type. Data are means ± SEM (*n* = 4). *p < 0.05, **p < 0.01, ***p < 0.001, ****p < 0.0001. eAMD, early age‐related muscle dysfunction; eAMD+Ex, early age‐related muscle dysfunction following 6–8 weeks of regular endurance exercise; HYA, healthy young adult.
**Figure S4**. Effects of aging and exercise on skeletal muscle mitochondria. (A) Representative immunoblot of ETC complex units: ATP5A (CV), UQCRC2 (CIII), SDHB (CII), NDUFB8 (CI), and COX IV (CIV) and quantification for each group. Proteins were normalized to Ponceau signal. (*N* = 4). (B) Total concentration of reduced glutathione (GSH) in GA lysates. Values normalized to milligrams of protein per well (*n* = 6–7). (C) Total concentration of oxidized glutathione (GSSG) in GA lysates. Values normalized to milligrams of protein per well (*n* = 6–7). (D) Ratio of GSH concentration to GSSG concentration in each sample (ANOVA *p* = 0.089). Data are means ± SEM; *p < 0.05, ***p < 0.001. eAMD, early age‐related muscle dysfunction; eAMD+Ex, early age‐related muscle dysfunction following 6–8 weeks of regular endurance exercise; HYA, healthy young adult.
**Figure S5**. Correlation analyses of significantly altered ultrastructural parameters and in vivo rate of muscle relaxation. Pearson correlation coefficient (*r*) and coefficient of determination (*r* ^*2*^) resulting from (A) Mean mitochondria area and relaxation, (B) Mean mitochondria circularity and relaxation, and (C) Mean MERC coverage and relaxation. (*n* = 3/group). eAMD, early age‐related muscle dysfunction; eAMD+Ex, early age‐related muscle dysfunction following 6–8 weeks of regular endurance exercise; HYA, healthy young adult.

Data S1.

Data S2.

Data S3.

Data S4.

Data S5.

Data S6.

Data S7.

Data S8.

## Data Availability

Proteomics data and analysis (i.e., GO and GSEA) are available in supplementary documents. All other data may be supplied upon request from the corresponding author.
