# Supplementary material for: Altered Relaxation and Mitochondria‐Endoplasmic Reticulum Contacts Precede Major (Mal)Adaptations in Aging Skeletal Muscle and Are Prevented by Exercise
Source: Aging Cell. 2025 Jun 30;24(9):e70137. doi: 10.1111/acel.70137 (PMC12419855; doi:10.1111/acel.70137)
Supplement: Supplementary file 1 — Figure S1. Phenotypic effects of aging in skeletal muscle. Refer to schematic in Figure 1A. (A) Bodyweight and wet muscle weight for (B) gastrocnemius, (C) tibialis anterior, and (D) plantaris muscles normalized to tibia length. (E) Maximal isometric torque of plantar flexors via stimulation (150 Hz) of the tibial nerve normalized to tibia length. (n = 4–12). (F) Percentage of different MyHC isoforms in PL muscle fibers (n = 3–6). (G) Mean MinFeret diameter of PL fibers considering all fiber types. (H) Mean MinFeret diameter of PL fibers separated by fiber type. Data are means ± SEM; *p < 0.05, **p < 0.01, ***p < 0.001, ****p < 0.0001. eAMD, early age‐related muscle dysfunction; eAMD+Ex, early age‐related muscle dysfunction following 6–8 weeks of regular endurance exercise; HYA, healthy young adult. Figure S2. Effects of regular endurance exercise on 21‐month‐old mice (eAMD+Ex). (A) Bodyweight before and after 6‐to‐8‐week treadmill intervention (n = 12). (B) Total running distance during treadmill exhaustion test (n = 12). (C) Percentage of initial force lost after 70 repetitive submaximal (50 Hz) stimulations (n = 12). Pre—before exercise regular exercise, Post—after regular exercise. Data are individual values; **p < 0.01, ****p < 0.0001. Figure S3. Effects of aging and exercise on plantaris fibers. (A) Mean MinFeret diameter of PL fibers separated by fiber type. Data are means ± SEM (n = 4). *p < 0.05, **p < 0.01, ***p < 0.001, ****p < 0.0001. eAMD, early age‐related muscle dysfunction; eAMD+Ex, early age‐related muscle dysfunction following 6–8 weeks of regular endurance exercise; HYA, healthy young adult. Figure S4. Effects of aging and exercise on skeletal muscle mitochondria. (A) Representative immunoblot of ETC complex units: ATP5A (CV), UQCRC2 (CIII), SDHB (CII), NDUFB8 (CI), and COX IV (CIV) and quantification for each group. Proteins were normalized to Ponceau signal. (N = 4). (B) Total concentration of reduced glutathione (GSH) in GA lysates. Values norm [file ACEL-24-e70137-s009.docx]

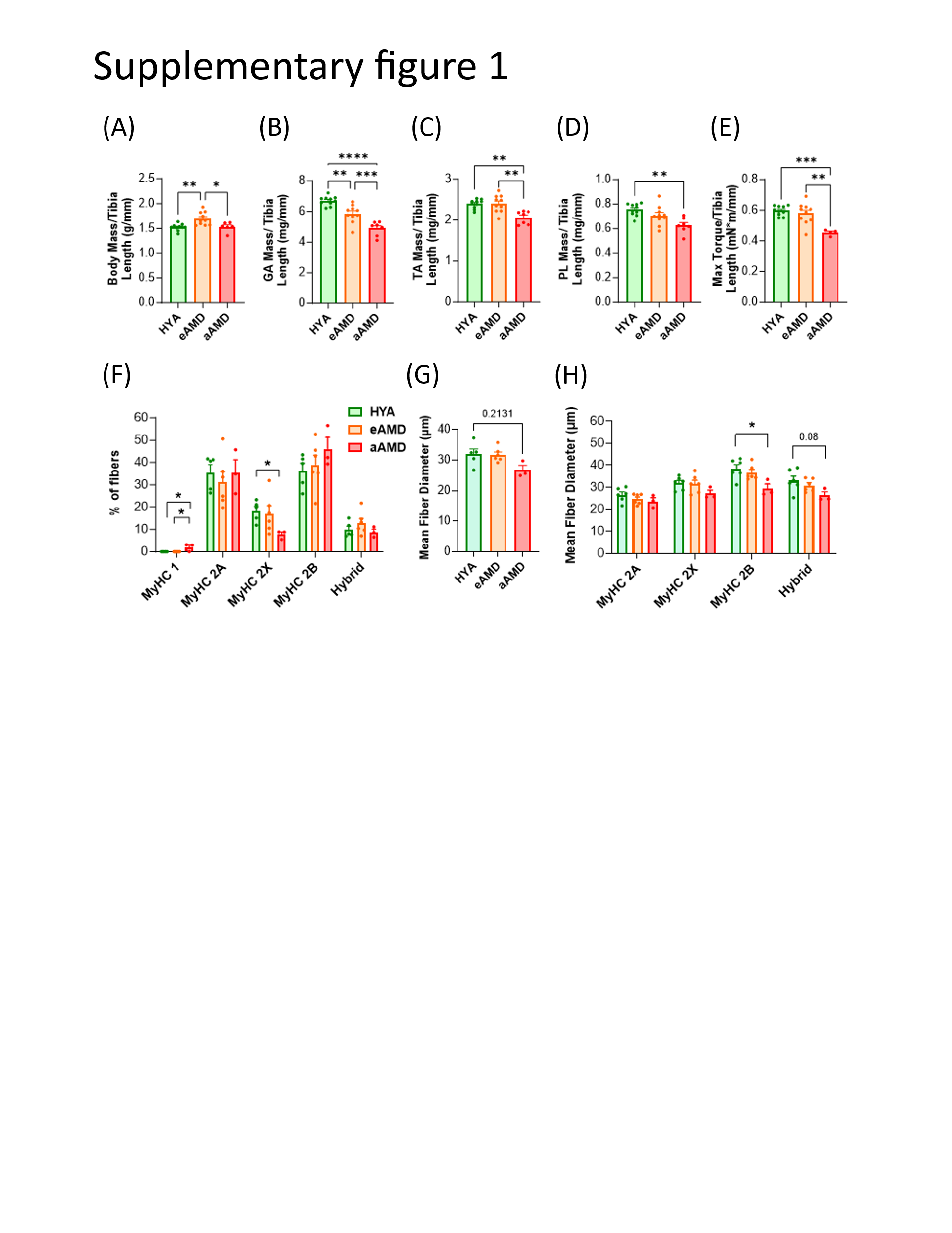


**Supplementary figure 1.** Phenotypic effects of aging in skeletal muscle. Refer to schematic in Figure 1A. (A) Bodyweight and wet muscle weight for (B) gastrocnemius, (C) tibialis anterior, and (D) plantaris muscles normalized to tibia length (*n* = 7-10). (E) Maximal isometric torque of plantar flexors via stimulation (150 Hz) of the tibial nerve normalized to tibia length (*n* = 4-10). (F) Percentage of different MyHC isoforms in PL muscle fibers (*n* = 3-6, which also applies to (G) and (H)). (G) Mean MinFeret diameter of PL fibers considering all fiber types. (H) Mean MinFeret diameter of PL fibers separated by fiber type. Data are means ± SEM; *p<0.05, **p<0.01, ***p<0.001, ****p<0.0001. HYA – Healthy Young Adult, eAMD – early Age-related Muscle Dysfunction, aAMD – advanced Age-related Muscle Dysfunction.


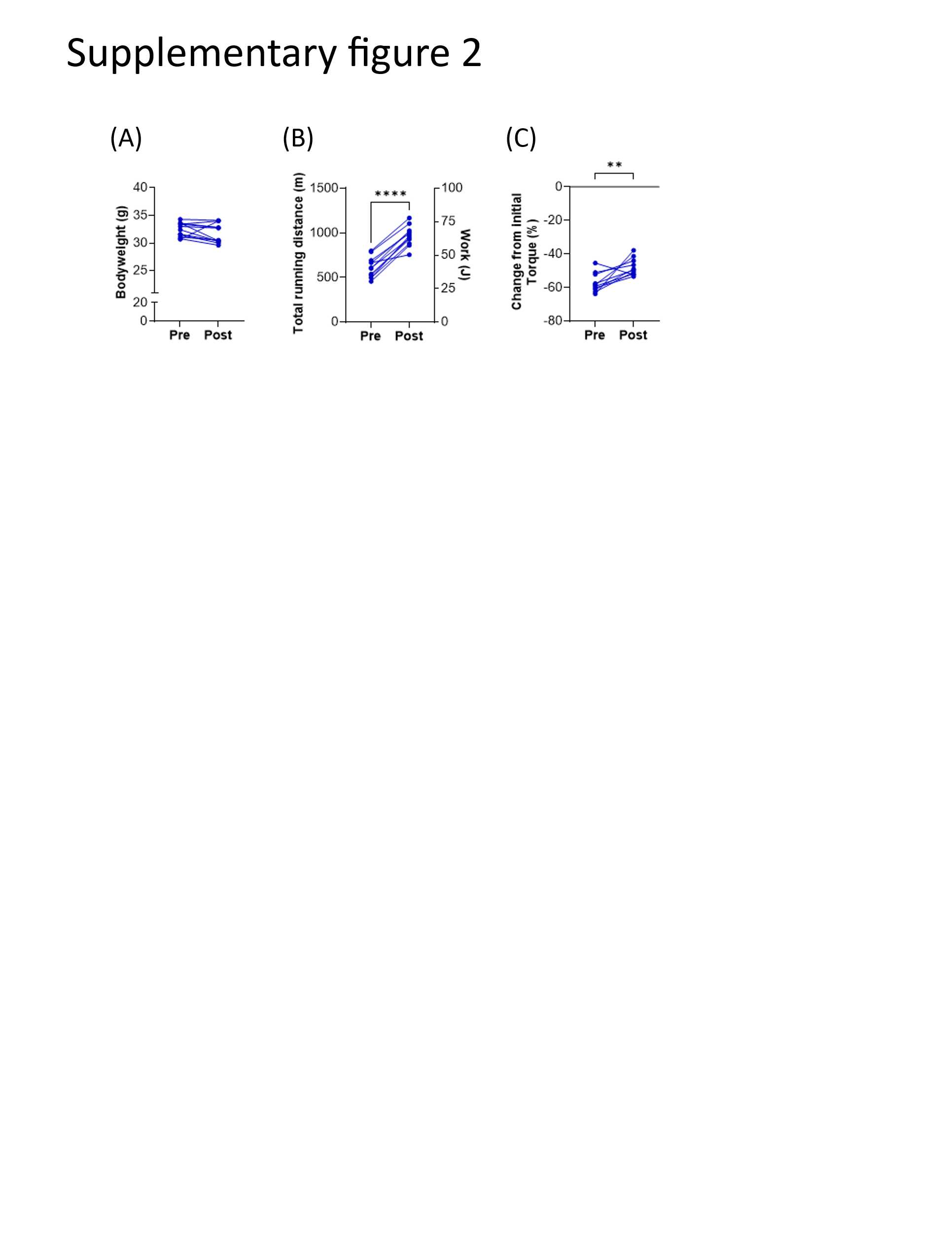


**Supplementary figure 2.** Effects of regular endurance exercise on 21-month-old mice (eAMD+Ex). (A) Bodyweight before and after 6-to-8-week treadmill intervention (*n* = 12). (B) Total running distance during treadmill exhaustion test (*n* = 12). (C) Percentage of initial force lost after 70 repetitive submaximal (50Hz) stimulations. (*n* = 12). Pre – before exercise regular exercise, Post – after regular exercise. Data are individual values; **p<0.01, ****p<0.0001.


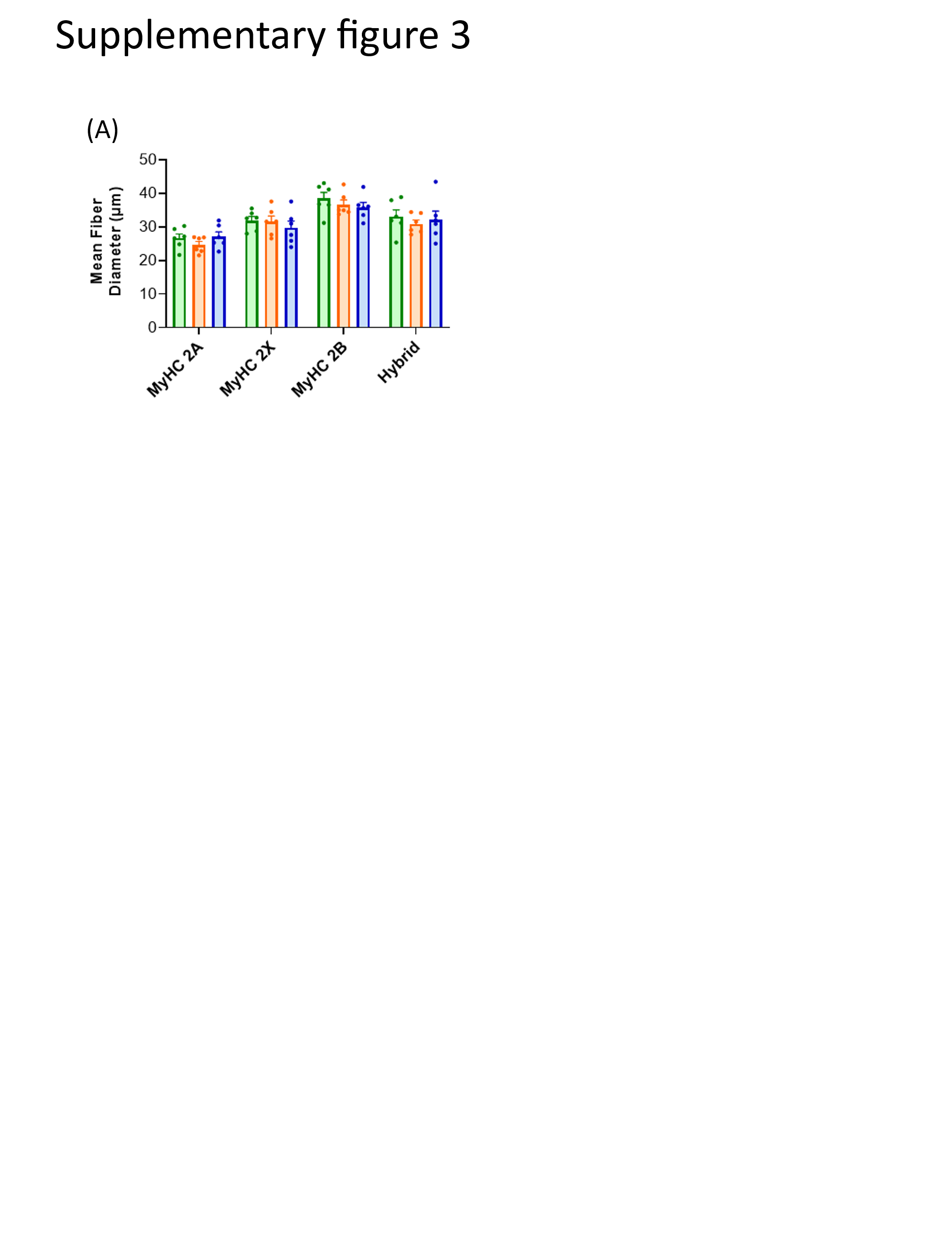


**Supplementary figure 3.** Effects of early aging and exercise on plantaris fibers. (A) Mean MinFeret diameter of PL fibers separated by fiber type. Data are means ± SEM (*n* = 6). HYA – Healthy Young Adult, eAMD – early Age-related Muscle Dysfunction, eAMD+Ex – early Age-related Muscle Dysfunction following 6-8 weeks of regular endurance exercise.


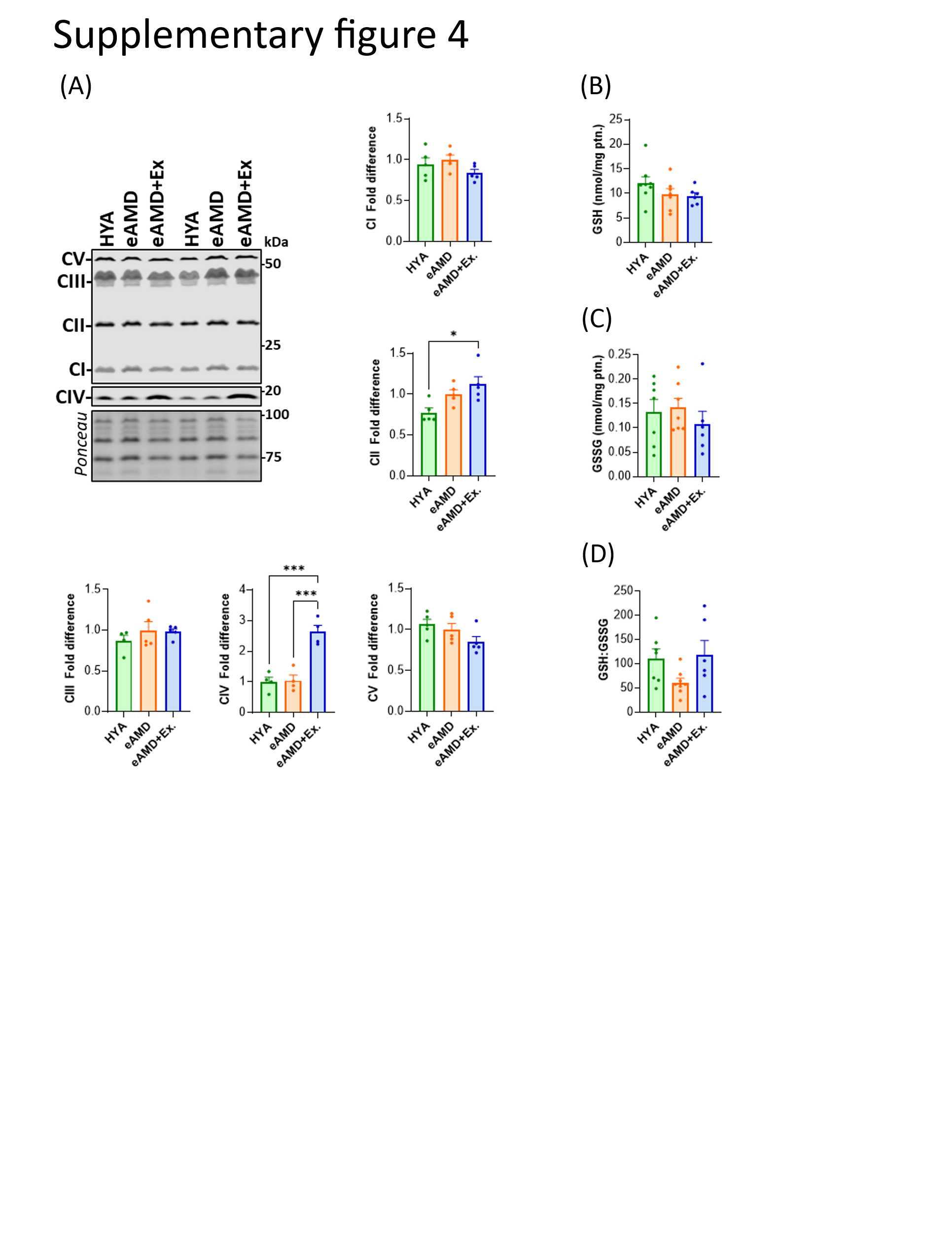


**Supplementary Figure 4.** Effects of aging and exercise on skeletal muscle mitochondria. (A) Representative immunoblot of ETC complex units: ATP5A (CV), UQCRC2 (CIII), SDHB (CII), NDUFB8 (CI), and COX IV (CIV), and quantification for each group. Proteins were normalized to Ponceau signal. (*n* = 5). (B) Total concentration of reduced glutathione (GSH) in GA lysates. Values normalized to milligrams of protein per well (*n* = 6-8, which also applies to (C) and (D)). (C) Total concentration of oxidized glutathione (GSSG) in GA lysates. Values normalized to milligrams of protein per well (*n* = 6-7). (D) Ratio of GSH to GSSG concentration in each sample (ANOVA p = 0.089). Data are means ± SEM; *p<0.05, ***p<0.001. HYA – Healthy Young Adult, eAMD – early Age-related Muscle Dysfunction, eAMD+Ex – early Age-related Muscle Dysfunction following 6-8 weeks of regular endurance exercise.


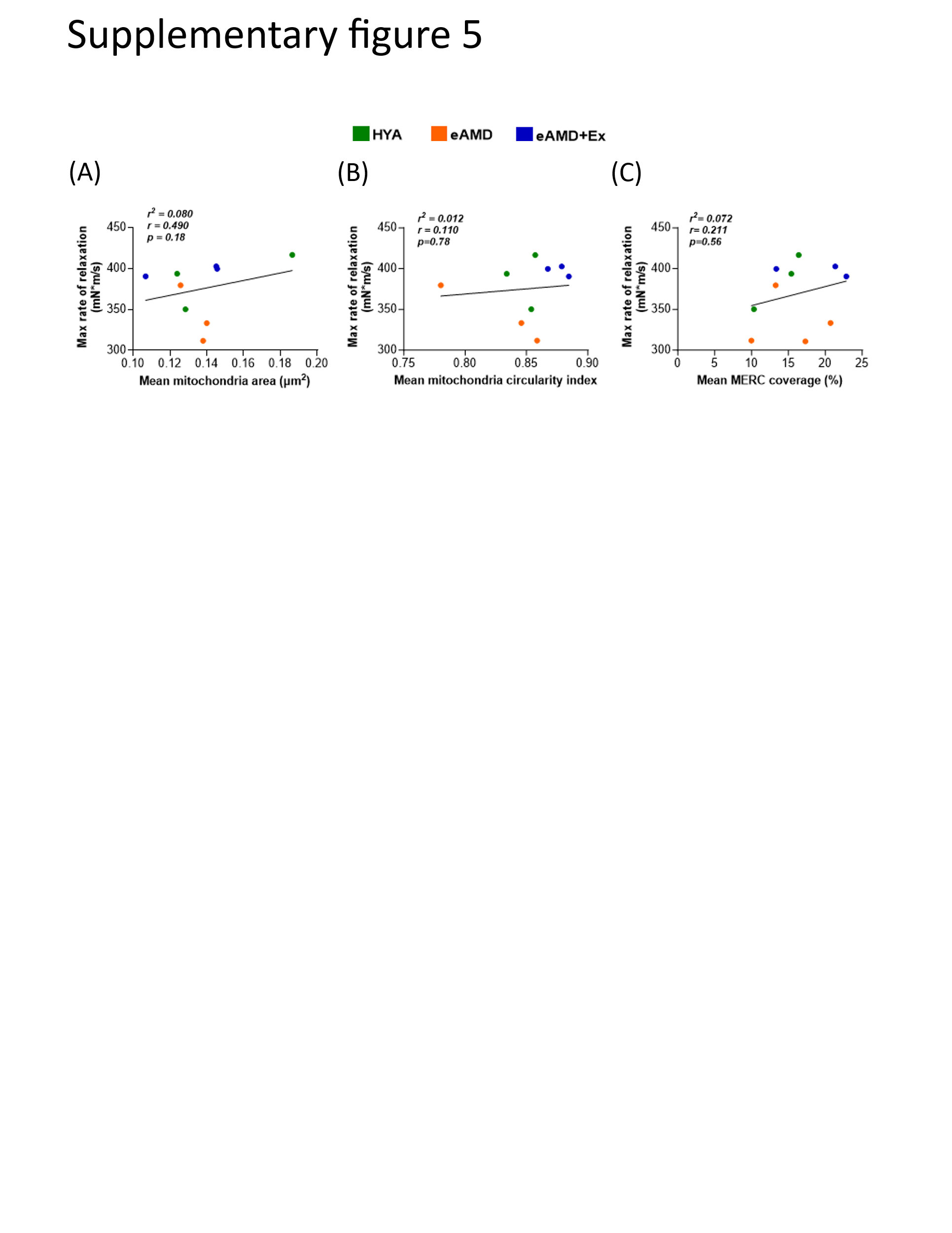


**Supplementary Figure 5**. Correlation analyses of ultrastructural parameters and *in vivo* rate of muscle relaxation. Pearson correlation coefficient (*r*) and coefficient of determination (*r^2^*) resulting from (A) Mean mitochondrial area and relaxation, (B) Mean mitochondrial circularity and relaxation, and (C) Mean MERC coverage and relaxation. (*n* = 3/group). HYA – Healthy young adult, eAMD –Early age-related muscle dysfunction, eAMD+Ex – Early age-related muscle dysfunction following 6-8 weeks of regular endurance exercise.
